# Peptide Aggregation Induced Immunogenic Rupture (PAIIR)

**DOI:** 10.1002/advs.202105868

**Published:** 2022-05-22

**Authors:** Gokhan Gunay, Seren Hamsici, Gillian A. Lang, Mark L. Lang, Susan Kovats, Handan Acar

**Affiliations:** ^1^ Stephenson School of Biomedical Engineering University of Oklahoma Norman OK 73069 USA; ^2^ Department of Microbiology and Immunology University of Oklahoma Health Sciences Center Oklahoma City OK 73104 USA; ^3^ Arthritis & Clinical Immunology Program Oklahoma Medical Research Foundation Oklahoma City OK 73104 USA; ^4^ Stephenson Cancer Center University of Oklahoma Health Sciences Center Oklahoma City OK 73104 USA

**Keywords:** damage‐associated molecular patterns, immunogenic cell death, influenza vaccination, peptide aggregation

## Abstract

Immunogenic cell death (ICD) arises when cells are under stress, and their membranes are damaged. They release damage‐associated molecular patterns (DAMPs) that stimulate and drive the type and magnitude of the immune response. In the presence of an antigen, DAMPs ride the longevity and efficacy of antigen‐specific immunity. Yet, no tool can induce the controlled ICD with predictable results. A peptide‐based tool, [II], is designed that aggregates in the cell and causes cell membrane damage, generates ICD and DAMPs release on various cell types, and hence can act as an adjuvant. An influenza vaccine is prepared by combining [II] with influenza hemagglutinin (HA) subunit antigens. The results show that [II] induced significantly higher HA‐specific immunoglobulin G1 (IgG1) and IgG2a antibodies than HA‐only immunized mice, while the peptide itself did not elicit antibodies. This paper demonstrates the first peptide‐aggregation induced immunogenic rupture (PAIIR) approach as a vaccine adjuvant. PAIIR is a promising adjuvant with a high potential to promote universal protection upon influenza HA vaccination.

## Graphical abstract

Peptide‐aggregation induced immunogenic rupture on the cell membrane induces the release of damage associated molecular patterns, which can activate the antigen presenting cells and adaptive immune response.

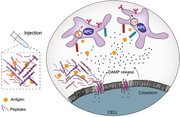

## 1 Introduction

As a result of cell membrane damage or stress, cells release damage‐associated molecular patterns (DAMPs) and die through immunogenic cell death (ICD), which has powerful immune system enhancing effects. Several infectious pathogens and cancers deploy strategies to limit the emission of DAMPs and escape immunosurveillance.^[^ ^1^ ^]^ Inducing and controlling localized ICD in the presence of an antigen has a significant potential for therapeutic and protective immunity. Released DAMPs with immunostimulatory effects show “adjuvant‐like” properties and engage antigen‐presenting cells (APCs) to stimulate a robust, long‐lasting immunity. In cancer treatment, inducing ICD and DAMP release with chemotherapeutics and photothermal therapy improves the antitumoral effect.^[^ ^2^, ^3^ ^]^ Similarly, engaging DAMP release enhances the antigen‐specific immunity in vaccination strategies.^[^ ^4^ ^]^ Despite this potential, the current ICD‐inducing technologies are limited because they are effective only in specific applications and demonstrate off‐target toxicity.^[^ ^5^, ^6^ ^]^ Therefore, there is an emerging clinical need for a tool that can induce controlled ICD and is effective across different cell types to increase the efficacy of current immunotherapy treatments.

Vaccination is critical for preventing the spread of respiratory viruses like influenza. However, current influenza vaccines have limited efficacy and cannot provide long‐lasting and universal protection due to the high mutation rate in influenza hemagglutinin (HA) antigens.^[^ ^7^, ^8^, ^9^ ^]^ Induction of a strong cellular immunity against more conserved antigens of influenza is critical for universal protection.^[^ ^10^ ^]^ However, vaccination with the influenza HA protein combined with the majority of the commercially available adjuvants have limited long‐lasting universal protection.^[^ ^11^ ^]^

Adjuvants induce antigen‐specific antibodies to either tag the antigens on pathogens or infected cells for attack by effector immune cells (as in type‐1 cellular response correlated to the CD4^+^ T cells called T helper 1, Th1) or neutralize antigen‐carrying entities (as in type‐2 humoral response linked to the CD4^+^ T cells called T helper 2, Th2).^[^ ^4^, ^12^ ^]^ Most common adjuvants enhance type‐2 response via Th2 and produce neutralizing antibodies, immunoglobulin G1 (IgG1). A typical Th1 response to a viral infection is characterized by recruitment of cytotoxic T lymphocytes (CTL), macrophages, NK cells, and is associated with IgG2a or IgG2c formation in mice. Particularly IgG2a production is driven by IFN‐γ, whereas IgG1 occurs through IL‐4.^[^ ^13^ ^]^ Indeed, the expression of these antibody type is used to identify the type of immune responses in mice.^[^ ^14^ ^]^

Naturally existing cell membrane‐disrupting proteins (or peptides) often generate stress and DAMP‐release through aggregation, such as perforin, gasdermins, or amyloid‐beta.^[^ ^15^, ^16^ ^]^ Induced membrane damage subsequently releases natural adjuvants called DAMPs, such as ATP, DNA, high‐mobility group box 1 (HMGB‐1), and heat shock protein 90 (HSP90).^[^ ^17^, ^18^ ^]^ Antigen presenting cells (APCs) recognize DAMPs through their pattern recognition receptors (PRRs) and become activated,^[^ ^19^ ^]^ generating non‐infectious local inflammation and enhancing their ability to internalize and present antigens to activate the adaptive immune response synergistically.^[^ ^20^ ^]^ For example, oil‐in‐water emulsion MF59 induced ATP release from muscle cells through intramuscular vaccination, and degradation of extracellular ATP reduced HA‐specific antibody formation when mice immunized with HA‐influenza antigens.^[^ ^21^ ^]^ However, ATP injection alone was insufficient to induce a strong response, suggesting a synergistic effect that relies on other released DAMPs.^[^ ^21^ ^]^ In a recent study, plasmid‐encoded HMGB‐1 increased the immunogenicity of a DNA vaccine, thus enhancing a specific immune response to the influenza nucleoprotein and HA proteins, and improving the survival of mice against lethal mucosal heterosubtypic influenza challenge.^[^ ^22^ ^]^ HSP90 enhances the cross‐presentation of antigens by APCs.^[^ ^23^ ^]^ However, recombinant DAMPs have been ineffective adjuvants.^[^ ^24^ ^]^ Therefore, technologies that induce the release of DAMPs collectively for synergistic effects are an emerging frontier in immunotherapy.

To induce such DAMP‐release with a peptide that targets a specific cell membrane receptor has limitations; peptide will not be effective on the cells without the receptor or cell can mutate to reduce the expression of that receptor to gain resistance against the peptide. Therefore, rather than targeting a particular protein, in this study, we utilized the co‐assembly of oppositely charged peptides (CoOP) strategy to design a peptide sequence that can aggregate strong enough in the cellular environment to induce similar ICD functionality.^[^ ^25^ ^]^

Nanomaterials built by self‐assembling peptides have broad application areas in medicine. Discovery of peptide sequences that assemble into nanostructures with desired properties and functions in a biological environment is a major challenge for the field. Editing the sequence of a protein that naturally assembles in the body can accelerate the discovery of various sequences, yet the harvested properties rule their functionalities. Establishing the application of a new peptide material based on its characterized properties is a common strategy.^[^ ^26^ ^]^ Here we applied a unique approach; instead of mimicking a naturally existing protein or peptide sequence, we aimed to mimic their function and activity with a new tool that induces ICD via CoOP strategy. We designed peptide sequences for mimicking this activity. CoOP strategy is based on a framework (i.e., the diphenylalanine (FF) domain and terminal charges) that defines the peptide‐peptide orientation and thus initiates the interactions of the peptides. However, the forces that affect the kinetics of the assembly and the properties of the final product are provided by the two amino acids of the substitution domain [XX] (we use the standard single‐letter amino‐acid codes throughout this work, with the CoOP pairs denoted in square brackets: [ ]). Our CoOP strategy provides a quantitative correlations between changes in the amino acid sequence of the framework and the properties of the assembly.^[^ ^25^ ^]^ Hence, CoOP represents a uniquely powerful strategy to create small peptides with controllable aggregation profiles that can be used to design peptide‐aggregation‐induced immunogenic rupture (PAIIR) of the cell membrane and subsequent ICD.

**Figure 1:**
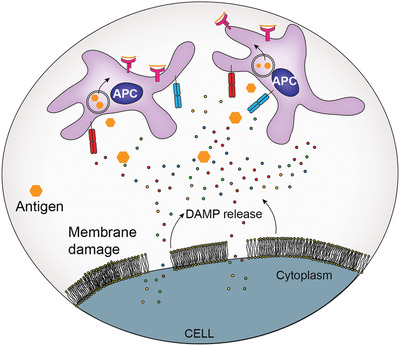
Cell membrane damage leads to the release of DAMPs. These local DAMPs activate antigen‐presenting cells for the uptake, processing and presentation of antigens.

Here, we hypothesize that PAIIR on cells can be utilized as a vaccine adjuvant. We developed an influenza vaccine and studied the immune response by mixing our new peptide tool with influenza HA proteins (**Figure** **1**). We demonstrate the use of the CoOP strategy to design peptide sequences with the desired aggregation properties to compose PAIIR. We identify the mechanism of programmed ICD and demonstrate DAMP release induced by our designed peptides in multiple cell types. Upon co‐administration, PAIIR enhances HA‐specific IgG1 and IgG2 formation significantly compared to immunization with HA alone. Therefore it is likely that PAIIR broadens the humoral immunity in mice. More importantly, we show that the designed peptide did not generate any specific antibody against itself. These results highlight PAIIR as a promising new tool that can readily be injected, providing a simple, efficient, and safe method for enhancing immune response in vaccine applications.

## 2 Results and Discussions

### 2.1 Designing Peptides as ICD Inducing Tools

**Figure 2:**
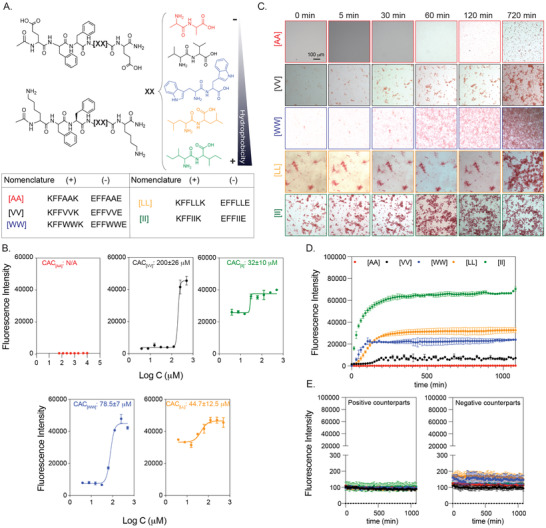
Peptide groups and aggregation kinetics. (A) CoOP groups and individual counterparts were used in this study. (B) CAC of co‐assembled peptides with DPH. (C) Macroscopic CoOP aggregates incubated with Congo Red. (D) Determination of aggregation kinetics of CoOPs and (E) individual counterparts by ThT assay. Data are representative of three experiments.

Engineering the fine‐tuned cellular aggregation of peptides requires well‐characterized intermolecular interactions.^[^ ^27^, ^28^ ^]^ Understanding these interactions allows the rational design of peptides to encompass desired properties, such as aggregation on the cell that induces stress for inducing ICD and cell membrane rupturing for DAMP release. We utilized the CoOP strategy to design peptides with strong enough affinity to aggregate in the cellular environment. The local charges in this design promote electrostatic interactions between two oppositely charged amino acids; the anionic carboxylate of Glutamic acid (E) and the cationic ammonium from Lysine (K) (**Figure** **2**A). The deliberately short, charged design enables the internalization of CoOP‐based peptides into cells.^[^ ^29^ ^]^ Design studies on multidomain peptides revealed the importance of hydrophobic amino acid localization in the core of the structures.^[^ ^30^ ^]^ In particular, the interplay between the backbone hydrogen bonding and hydrophobic interactions among amino acid side‐chains is crucial to the formation of fibrillar structures.^[^ ^31^ ^]^ Therefore, to change the aggregation kinetics, we studied the hydrophobic amino acids in the substitution domain (“[XX]”), with increasing hydrophobicity indices; [AA] < [VV] < [WW] < [LL] < [II] (Figure 2A).

Characterization assays were performed in 1XPBS to mimic the salt concentration in the physiological conditions. The critical aggregation concentrations (CAC), that is, the minimum concentration needed for co‐assembly of the peptides, are measured with DPH ((1,6‐diphenyl‐1,3,5‐hexatriene)), which becomes fluorescent when located in aggregates with hydrophobic cores.^[^ ^34^ ^]^ The CAC values of [VV]=200±26 µm, [WW]=78.5±7 µm, [LL]=44.7±12.5 µm, and [II]=32±10 µm followed the same trend as the hydrophobicity of the amino acids at their core (Figure 2B). The identified CAC values were correlated to our previous study.^[^ ^25^ ^]^. No fluorescence was observed for [AA] even at 10 mm, indicating no aggregation; therefore, this situation represents an excellent control for studying the effects of peptide aggregation, as the same concentration of peptides with similar sequences and charges will remain free components in solution.

The aggregation kinetics of the peptide pairs were measured with Congo Red and Thioflavin T (ThT) assays. Congo Red staining has been used to identify amyloid fibrils in vitro and tissue sections, emitting red light due to the binding of β‐sheet rich domains, leading to a redshift in its absorbance peak from 490 to 512 nm.^[^ ^35^ ^]^ We monitored the fluorescence of Congo red for 720 min (12 h) (Figure 2C) at 0.5 mm peptide concentration. [AA] precipitation appeared after 720 min; these are not likely to be ordered aggregates with hydrophobic cores because no DPH fluorescence was observed at this concentration. [II] aggregated instantaneously (0 min), while [LL] aggregates were deposited initially and became stable until 720 min. [WW] and [VV] did not show any initial aggregates but deposited after 30 min and 120 min, respectively. Despite the ease of visualization with red light, Congo Red is not as sensitive as ThT in understanding ordered structures since fluorescence methods (rather than absorption) are preferred for high‐sensitivity detection.^[^ ^36^ ^]^ Therefore, we use Congo Red as a visualization method, with ThT used for quantitative temporal measurements of oppositely charged pairs and each counterpart individually for 1200 min (20h). To determine the time needed for equilibrium assembly of CoOPs, we applied curve fitting to the ThT analysis (Figure 2D). [AA] did not assemble and thus did not show any fluorescence in ThT. For the remaining peptide pairs, the time to reach equilibrium assembly was calculated as [VV] (660 min) > [WW] (675 min) > [LL] (630 min) > [II] (510 min). Individual counterparts did not produce any fluorescence signal (Figure 2E). We observed that the like‐charged groups alone did not aggregate, highlighting the importance of electrostatic attractions in the aggregation process (Figure 2D). Among the studied peptides, [II] showed the fastest aggregation and the highest fluorescence intensity, indicating that [II] has the highest affinity to each other, and these peptides are stacked in a more well‐ordered structure than the other CoOPs. The TEM images of the aggregates showed one‐dimensional structures (Figure S5, Supporting Information). The use of the FF group in the CoOP sequence was for enhancing the one‐dimensional aggregation; thus, the observed structures were expected. These results indicate that control over peptide aggregation is achieved by changing the hydrophobicity of amino acids in the substitution domain.

### 2.2 Cell Viability

**Figure 3:**
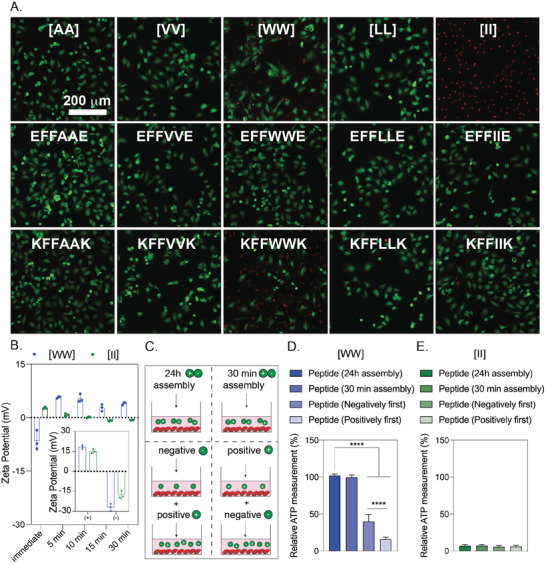
Cytotoxicity of peptides to OVCAR‐8 cells. Negative peptides were added to the cells, followed by corresponding positive peptides. A) Live‐dead images of OVCAR‐8 cells treated with peptide combinations and corresponding individual counterparts at 6h (peptide concentration is 0.5 mm). B) Zeta potential of individual and combined peptides. C) Illustration of peptide preparation for [WW] and [II]. The effect of peptide preparation on cell viability for [WW] D) and [II] E). The scale bar is 200 µm. Data are representative of at least three experiments. Statistical analysis via one‐way ANOVA test, data are mean ± SD, ****p < 0.0001.

We used the human ovarian cancer cell line OVCAR‐8 as our initial model to identify the effect of the peptides on cells. All cell culture experiments were performed in an RPMI cell culture medium with 10% FBS. At 0.5 mm (i.e., above the overall CAC), the individual peptides mixed on the cells by promptly adding the first negative, then positive counterparts. The viability of the cells was measured for 6h through live‐dead imaging (**Figure** **3**A). Among the peptide groups studied, [AA], [VV], and [LL] did not affect cell viability in the measured time frame (Figure 3A). Only [WW] and [II] caused cell death compared to the control group. Because the peptides, if aggregate, form one‐dimensional structures, the shape of the aggregates is not likely to be effective on the action of the peptides. [II] has the lowest CAC and highest ordered aggregation (Figure 2B–D). Additionally, the equilibrium time of [II] aggregations was the shortest among the tried groups (**Table** **1**). These all indicate that [II] peptides have the highest affinity to each other, which creates aggregations strong and fast enough to induce cell death.

**Table 1:** The relationship of side‐chain properties with CAC and equilibrium time for co‐assembly of CoOPs

	[AA]	[VV]	[WW]	[LL]	[II]
CAC [µm]	N/A	200±26	78.5±7	44.7±12.5	32±10
Equilibrium reaching time [min]	N/A	690	675	630	510
Side‐chain hydrophobicity^[^ ^32^ ^]^	41	76	97	97	99
Hydrophobic/hydrophilic surface area^[^ ^33^ ^]^	1.5	3.1	2.3	3.6	3.9
t1/2 (half time, min)	N/A	96.3±10.8	33.6±1.9	79.4±3.6	53.45±1.82

To understand the effect of peptide aggregates versus peptides alone on the cell death identified with [WW] and [II], we performed a live/dead assay with individual peptides at 0.5 mm after 6h (Figure 3A). Despite the dramatic cell death observed with the [II] mixture at this concentration, the individual [II] peptides did not induce cell death. Similarly, the negative [WW] (EFFWWE) peptide alone did not show any cell death, yet the positive [WW] (KFFWWK) peptide induced the cell death (as indicated by red‐stained cells). Positive charged peptides containing W are promiscuous residues for membrane damaging peptides, possibly because of W's “anchoring” role; W is abundant in membrane proteins, particularly near the lipid–water interface.^[^ ^37^, ^38^ ^]^ Furthermore, as analyzed by zeta‐potential, individual peptides showed the expected overall charges at pH 7.0 ((pKa of (‐COO^‐^) of E is 4.25 and (‐NH_3_^+^) of K is 10.53) (Figure 3B, inset). In an aqueous solution, the [II] aggregates acquire a neutral charge in 5 min, while [WW] showed a slight positive charge after 30 min, possibly due to incomplete assembly (Figure 3B).

Given the effects of individual [II] and [WW] on cell viability, we examined the order of addition of the peptide counterparts; first positive and then negative peptide, and vice versa (Figure 3C). The addition of KFFWWK first induced significantly higher cell death (****p < 0.0001) compared to the initial addition of negative peptide, indicating that the cell death is due to the positive charge of KFFWWK (Figure 3D). Yet, any order change did not affect the membrane damage activity levels for [II] (Figure 3E). Moreover, mixing the peptides for 30 min or 24h before addition to the cells completely abolished the effect of [WW] (Figure 3D), indicating that the initial membrane damage was due to the positive charge of KFFWWK which was neutralized upon mixing and aggregation with its counterpart (Figure 2B). Nevertheless, the pre‐mixing did not alter the effect of [II] under these conditions (Figure 3E), highlighting that [II] is the only peptide pair among those studied that induces cell death through the aggregation of its charged counterparts. Although these charges help peptides find each other even in low concentrations, the electrostatic interactions do not contribute to the stability or thermodynamic assembly of the peptides, which happens only through the hydrophobic amino acids in the core of the sequence.^[^ ^25^ ^]^ Our results show that increasing the hydrophobicity of the amino acid decreases the aggregation time, which has a direct effect on cell membrane damage. Isoleucine is the most hydrophobic canonical amino acid, and [II] peptides have the highest affinity among the studied pairs and showed the lowest CAC in the shortest aggregation equilibrium time (Figure 2). The affinity among [II] creates strong aggregates to induce ICD, which is the functionality that we aimed to mimic.

Synthetic peptides derived from natural proteins induce ICD through perturbation of both cell and mitochondrial membranes.^[^ ^39^, ^40^, ^41^ ^]^ However, these peptides target Bcl‐2 family proteins to create mitochondrial damage.^[^ ^42^, ^43^ ^]^ The oncogenic mutations in the Bcl‐2 proteins and known drug resistance against therapeutics targeting them, diminishes the effectiveness of these peptides.^[^ ^44^, ^45^ ^]^

### 2.3 Cell Membrane Rupture through Peptide Assembly

**Figure 4:**
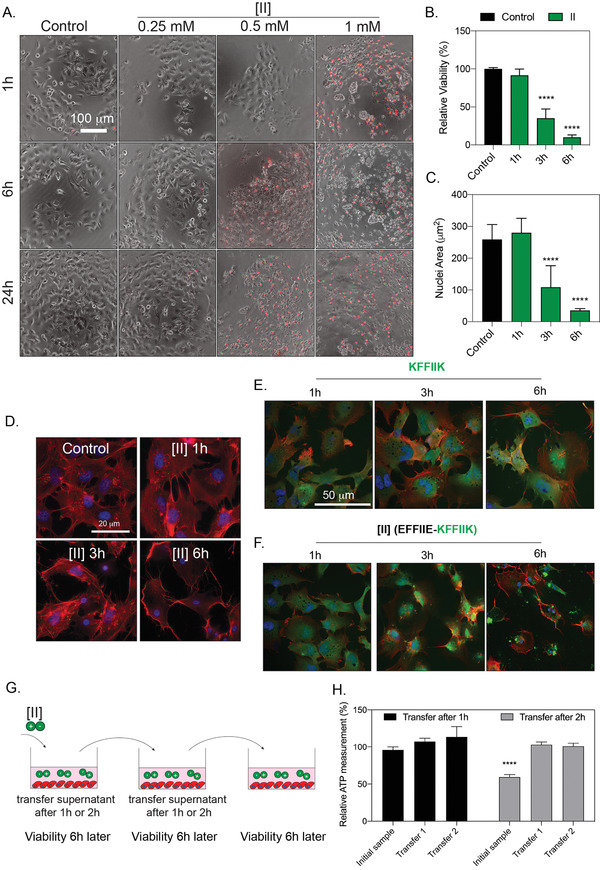
[II] induces membrane rupture. A) Time and concentration‐dependent cell membrane damage of [II] were analyzed through PI uptake (scale bar is 100 µm). B) Time‐dependent viability measurement and C) time‐dependent nuclear size analysis. D) Actin cytoskeleton staining of [II] treated OVCAR‐8 cells at 1,3 and 6h, scale bar is 20 µm. E) Internalization of FITC‐KFFIIK and F) FITC‐[II] for 1, 3 and 6h, scale bar is 50 µm. G) Experimental plan of [II] transfer and viability experiment. H) Effect of [II] transfer on cell viability at 6h. Data are representative of at least three experiments. Statistical analysis was done with one‐way ANOVA test. Data are mean ± SD, ****p < 0.0001.

The aggregation‐induced membrane damage activity was only observed with [II]; we analyzed how it induces cell death. We used propidium iodide (PI) to understand cell membrane damage, a dye that does not permeate intact cell membranes.^[^ ^46^ ^]^ **Figure** **4**A shows cells treated with three concentrations of [II]: 0.25 mm, 0.5 mm, and 1 mm for 1, 6, and 24h. Increasing the concentration to 1mm resulted in faster membrane damage, while decreasing the concentration to 0.25 mm (although still higher than the [II] CAC of 38 µm) did not have sufficient aggregation to trigger membrane damage in this time frame. The time and concentration dependence membrane damage activity of [II] is likely to result from faster aggregation in higher concentrations. These results show that [II] induced cell death can be controlled through time and concentration. Flow cytometry results of PI uptake were correlated with the imaging analysis showing >80% cell death at 6h for 0.5 and 1 mm [II] peptide treated cells (Figure S2A,B, Supporting Information). To understand the lowest [II] peptide concentration that can induce membrane damage, we treated the cells with 0.1‐0.2‐0.3‐0.4 and 0.5 mm [II] peptides. For 6 and 24h the lowest concentration resulting in membrane damage was 0.4 mm (Figure S3, Supporting Information).

The confocal images analyses of 0.5 mm [II], which induces > 85% of death in 6h (Figure 4B), showed cell swelling and nuclear condensation; hallmarks of necrotic cell death (Figures 4C and 4D).^[^ ^47^ ^]^ Aggregation was visualized by labeling the positive charged [II] (KFFIIK) peptide with FITC (green fluorescent). We monitored the internalization and aggregation of KFFIIK through fluorescence microscopy. KFFIIK alone was internalized into the cells starting at 1h (Figure 4E), and cellular morphology did not change over time as KFFIIK alone does not cause cell death. When KFFIIK was introduced with its negatively charged counterpart, EFFIIE, the peptide‐aggregation (more localized green KFFIIK) was apparent around and within the cells starting at 3h (Figure 4F). The peptide‐aggregation induced nuclear area shrinking and loss of integrity of the cells (observed through the absence of actin fibers), indicating [II] aggregation‐induced cell membrane damage and death.

Observing cell death beyond the desired area (i.e., off‐site cytotoxicity) is a major obstacle for existing therapeutic applications. To estimate the off‐site cytotoxicity of our approach, we incubated cells with 0.5 mm [II] for 1h or 2h, followed by the transfer of supernatant into another well with fresh cells, and measured viability after 6h (Figure 4G). We observed the membrane damage activity only in the initial sample after 2h (Figure 4H), suggesting that at this concentration, [II] does not induce cell membrane damage in 1h (in line with the viability results Figure 4B). Also, subsequent transfer of the supernatant of cells incubated with [II] for 1h did not induce cell membrane damage, possibly due to aggregation having started within the cells and thus insufficient [II] transfer to the next well.

### 2.4 [II] Aggregation‐Induced Cell Death is Immunogenic

**Figure 5:**
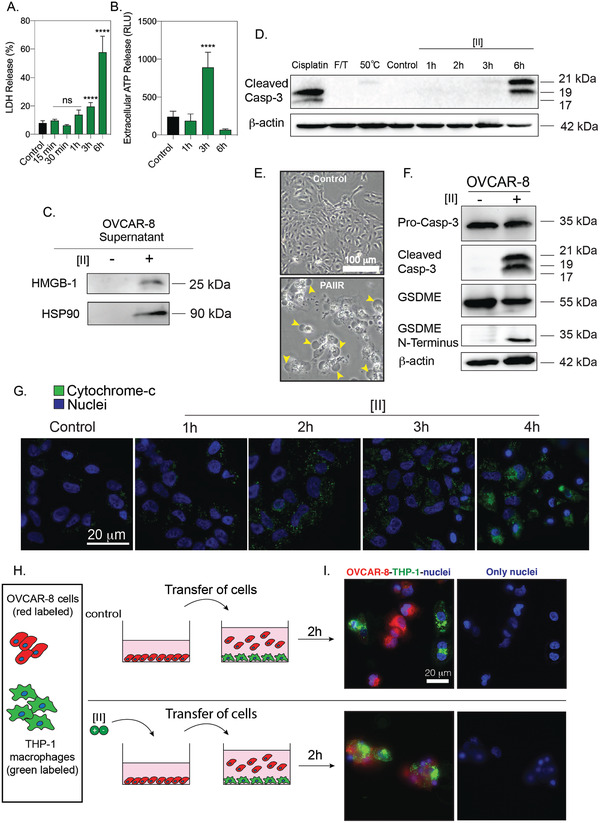
[II] induces DAMP release and secondary pyroptosis. A) Time‐dependent LDH release. B) Time‐dependent extracellular ATP release. C) Western blot analysis of HMGB‐1 and HSP90 release at 6h. D) Caspase‐3 cleavage after treatment with Cisplatin, F/T, heat treatment and 1‐2‐3 and 6h of [II] treatment. E) Phase‐contrast images of control and [II] treated OVCAR‐8 cells at 6h, yellow arrows indicate bubbles emerging from pyroptotic cells. The scale bar is 100 µm F) Caspase‐3 and GSDME cleavage of OVCAR‐8 cells with and without treatment with [II]. G) Time‐dependent cytochrome c release into the cytoplasm from OVCAR‐8 cells. The scale bar is 20 µm. H) Schematic illustration of the phagocytosis assay. I) Phagocytosis of OVCAR‐8 cells by THP‐1 differentiated macrophages after 6h of [II] and control treatment. The scale bar is 20 µm. Data are representative of at least three experiments. Statistical analysis via one‐way ANOVA test, data are mean ± SD, ****p < 0.0001.

DAMP release through cell membrane rupture is a hallmark of ICD. We measured the release of lactate dehydrogenase (LDH), a cytoplasmic enzyme released into the extracellular matrix upon membrane damage.^[^ ^48^ ^]^ LDH release became significant 3h after incubation with [II] (**Figure** **5**A), in agreement with the reduction of cell viability (Figure 4B). As expected, significant DAMP release followed the cell membrane damage, such as the presence of extracellular ATP (****p < 0.0001) after 3h (Figure 5B), correlated to the LDH release data. However, the amount of extracellular ATP is depleted in 6h due to cell death > 85% (i.e., few live cells remain to release ATP upon ICD) and rapid extracellular hydrolysis of ATP.^[^ ^49^ ^]^ Similarly, the release of other DAMPs (specifically HMGB‐1 and HSP90) was also detected in the extracellular environment of [II] treated cells at 6h via western blotting (Figure 5C). Our DAMP release profile results highlight that the observed cell death is immunogenic and induced by [II] peptide pair aggregation. In other words, peptide‐aggregation induced immune rupture (PAIIR) is demonstrated with the [II] pair.

Regulated cell death via an inducer is concentration and time‐dependent and therefore controllable. On the other hand, unregulated cell death cannot be controlled to the same extent as it mainly results from mechanical forces or temperature. The unregulated cell death releases an instantaneous burst of DAMPs, while the DAMP release from regulated cell death is prolonged. Therefore, regulated ICD is desired for a controlled immune response. Regulated necrotic cell deaths are caspase‐dependent.^[^ ^50^, ^51^ ^]^ To identify whether [II]‐induced cell death is regulated, we analyzed the cleavage of caspase‐3, a known mediator of apoptosis, and GSDME mediated pyroptosis (regulated necrosis).^[^ ^2^, ^52^ ^]^ We compared the caspase‐3 cleavage among different cell death modalities; we used cisplatin ‐ a known apoptosis inducer in the OVCAR‐8 cell line.^[^ ^53^ ^]^ We also used heat treatment to induce multiple cell death modalities, including necrosis, apoptosis, and necroptosis.^[^ ^54^ ^]^ Additionally, we applied a freeze/thaw (F/T) method, a common technique to induce unregulated necrosis.^[^ ^55^ ^]^ Figure 5D shows western blotting of caspase‐3 cleavage upon these treatments. Cisplatin showed caspase‐3 cleavage, which is expected in apoptotic cell death. Conversely, F/T did not show any caspase‐3 cleavage. Observation of cleaved caspase‐3 in [II] induced cell death indicates regulated necrosis (e.g., GSDME mediated pyroptosis).^[^ ^56^ ^]^ Indeed, pyroptosis can be identified via cleavage of caspase‐3 and GSDME and a ballooning morphology of the cell membrane.^[^ ^57^ ^]^ As cell membrane damage is already identified, we examined the morphological features of [II] treated cells and observed ballooning of the plasma membrane (Figure 5E, yellow arrows). At the molecular level, cleavage of caspase‐3 activates GSDME cleavage, then the N‐termini of the cleaved GSDME oligomerize and induces membrane pore formation.^[^ ^17^ ^]^ Western blot analysis showed that OVCAR‐8 cells express GSDME and treatment with [II] results in its cleavage at 6h (Figure 5F). These results show that [II] treated cells have the morphological and molecular features of GSDME‐mediated pyroptosis, a regulated ICD.

The majority of caspase activation within mammalian cells is initiated through cytochrome c, a protein normally found in mitochondria.^[^ ^58^ ^]^. Released cytochrome c initiates the activation of caspase‐3^[^ ^59^ ^]^ and the downstream pathway of cytochrome c release is not dependent on the functions of BAX or BAK proteins.^[^ ^43^ ^]^ Furthermore, cytoplasmic cytochrome c release has been shown to lead to the induction of GSDME‐mediated pyroptosis in cells that express GSDME.^[^ ^57^ ^]^ To understand the origin of caspase‐3 cleavage upon [II] treatment, we stained cytoplasmic cytochrome c to analyze mitochondrial damage as shown before.^[^ ^60^ ^]^ Time‐dependent imaging showed that cytochrome c levels increased over time in the cytoplasm of OVCAR‐8 cells (Figure 5G), specifically at 4h, explaining the abundance of cleaved caspase‐3 at 6h (Figure 5D). These results show the general mechanism of PAIIR; [II] peptide directly damages the cell membrane, as the LDH release was observed in the first 3h of the incubation along with the ATP release (Figure 5A,B), yet the caspase 3 cleavage was abundant after 3h (Figure 5D). [II] peptide induces plasma and mitochondrial membrane damage, leading to cytochrome c release into the cytoplasm; this release activates caspase‐3 cleavage, and initiates GSDME‐mediated pyroptosis.

ICD and DAMP release is known to enhance the phagocytosis of APCs.^[^ ^55^ ^]^ We checked whether [II] treated cells are phagocytosed by macrophages. THP‐1 differentiated macrophages are commonly utilized for in vitro phagocytosis experiments^[^ ^61^ ^]^. We treated red‐labeled OVCAR‐8 cells for 6h with [II] and co‐cultured them with green‐labeled THP‐1 macrophages for 2h. We observed phagocytosis of [II] treated cells by the macrophages, yet control cells were not phagocytosed (Figure 5I).

### 2.5 Peptide Assembly Induced ICD in Different Cell Types

**Figure 6:**
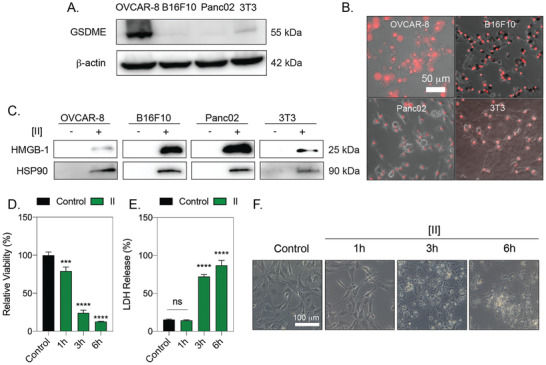
[II] induced membrane rupture and DAMP release is not cell type‐specific. A) GSDME analysis of OVCAR‐8, B16F10, Panc02 and 3T3 cells. B) PI uptake at 6h of [II] treatment on cells. The scale bar is 50 µm C) HMGB‐1 and HSP90 release to the supernatant (SN) at 6h. D) Time dependent viability measurement. E) Time‐dependent LDH release. F) Phase‐contrast images of [II] treated fibroblasts at 1, 3 and 6h. The scale bar is 100 µm. Data are representative of at least three experiments. Statistical analysis was done by one‐way ANOVA test, data shown are mean ± SD,****p < 0.0001, ***p < 0.001.

Induction of pyroptosis through raptinal (small drug) was shown to convert to apoptosis in the absence of GSDME, which is immunologically silent.^[^ ^62^ ^]^ To identify whether [II] induced ICD requires GSDME expression, we tested various cell lines with different GSDME expression profiles; B16F10 and Panc02 cells lines showed no GSDME expression, while 3T3 fibroblasts had low expression levels compared to OVCAR‐8 (**Figure** **6**A). Incubation of 0.5 mm [II] showed a similar cell membrane rupture effect with these cell lines, as indicated by PI uptake (Figure 6B). [II] initiated the extracellular release of DAMPs; HMGB‐1 and HSP90 in 6h (Figure 6C). Bubbling cell membrane structures were not observed in the cells that lacked GSDME (Figure 6A), suggesting that pyroptosis did not occur. These results show that [II] induced ICD is not dependent on GSDME, although it also has a GSDME‐regulated secondary mechanism. Expression of GSDME was linked to anti‐tumor immunity and tumor suppression.^[^ ^62^ ^]^ However, GSDME is expressed at low levels in most cancer types^[^ ^63^ ^]^ due to epigenetic silencing through methylation.^[^ ^64^ ^]^

One of the cell types we analyzed was fibroblasts, an abundant cell type in the subcutaneous layer.^[^ ^65^ ^]^ After subcutaneous vaccination, the stimulation of DAMP release from fibroblasts would act as natural adjuvants. Therefore, we quantified the [II] induced ICD of 3T3 fibroblasts with time‐dependent cell death and LDH release (Figure 6D–F), and the effect of [II] on fibroblasts was similar to that on OVCAR8.

Observation of ICD in various cell lines makes PAIIR an excellent adjuvant candidate. Although the mechanisms of clinically available adjuvants have not been entirely elucidated, the local cell death induction is a known part of their mechanism.^[^ ^4^ ^]^ DAMP release from local healthy cells has been shown to be the mechanism of action for commonly utilized adjuvants. MF59 stimulates ATP release from muscle cells^[^ ^21^ ^]^ while Alum has been shown to stimulate IL‐33 from alveolar epithelial cells.^[^ ^66^ ^]^

### 2.6 [II] Induced Immune Response Enhances the Specific Antibody Formation against Influenza Antigens

**Figure 7:**
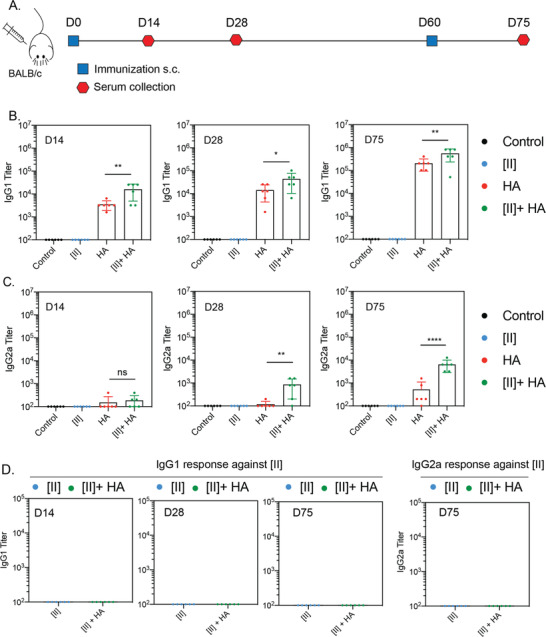
PAIIR induces influenza‐antigen specific antibody formation. A) Timeline and the plan of the vaccination study: BALB/c mice were vaccinated at D0 followed by a booster at D60, serum was collected at D14, 28 and 75. B) Antigen‐specific serum IgG1 titers at D14, 28 and 75. C) Serum IgG2a titers at D14, 28 and 75. D) Serum IgG1 and IgG2a titers against [II] peptide at D14, D28 and D75. Statistical analysis was done by one‐way analysis of variance (ANOVA) with Tukey's multiple‐comparison test, data shown are mean ± SD ****p < 0.0001, **p < 0.01, *p < 0.1 n = 6 mice.

We tested the hypothesis that the DAMP release caused by [II], in the presence of HA antigen, can enhance the antibody responses to influenza HA (**Figure** **7**A). Balb/c mice were immunized with PBS vehicle, recombinant HA, [II], or HA + [II]. After 60 days, mice received booster vaccines identical to the initial vaccine dose. Mice were bled at two‐time time points after the primary immunization (days 14 and 28) and once after the booster vaccine (day 75). HA‐specific IgG1 could not be detected following administration of PBS vehicle or [II] alone (Figure 7B). HA‐specific IgG1 was detected and increased with time since the primary immunization and was increased over 10 fold by the booster vaccine. At each of these time points, the addition of [II] in the formulation caused a 2‐4 fold increase in HA‐specific IgG1 titers. A similar pattern was observed for the less abundant IgG2a subclass, where [II] HA exerted a strong and significant adjuvant effect on the HA‐specific IgG2a titer (Figure 7C). Although the overall IgG response remained IgG1‐dominated, by day 28, [II] had increased HA‐specific IgG2a titers 7.3 fold and 12.4 fold by day 75. Consistent with the observation that [II] alone did not induce HA‐specific IgG1 or IgG2a responses, with the ELISA plates were also coated with [II] (Figure 7D). The skin where the [II] injection was performed subcutaneously did not show any external signs of inflammation (Figure S4, Supporting Information). These results, therefore, demonstrate that [II]‐induced ICD and DAMP‐release in the body can create an adjuvant effect when injected with HA. In the previous studies, IgG2a formation in mice was shown to generate higher cross‐protection (heterosubtypic) against lethal challenge with different influenza virus strains.^[^ ^12^, ^67^, ^68^ ^]^ Given the need for a universal influenza vaccine, any adjuvant that can boost the IgG2a response could contribute to achieving that goal. A challenge study using different influenza strains, including the heterosubtypic cross‐protection and comparison of the clinical adjuvants are the next steps for PAIIR to explore its protective properties.

The lack of an [II]‐specific IgG response is informative. Professional APCs express the Major Histocompatibility Complex (MHC) class II. Loading of MHC molecules with self and foreign antigen‐derived peptides governs immunological tolerance and the immune response to pathogens, respectively. The peptide‐binding groove of MHC‐II accommodates peptides of 13‐25 amino acids.^[^ ^69^ ^]^ The B cell antigen receptor (BCR) recognizes linear and conformational epitopes consisting of peptide sequences or other macromolecules. Because [II] is a peptide‐based adjuvant, the possibility of MHC‐II presentation or recognition by the BCR was considered. Our results showing no antibody response against [II] itself indicate that it is unlikely that the hexameric [II] peptide can bind MHC‐II or be recognized by the BCR.

## 3 Conclusions

Interest in peptide‐based materials has flourished in health, energy, materials science, and national security.^[^ ^27^ ^]^ By advancing the discovery of peptide domains with unique intermolecular interactions, the design of peptides with desired properties in appropriate conditions can be achievable. Inspired by the mechanism of natural membrane rupturing proteins that aggregate and induce ICD, in this study, we designed a new peptide‐based tool, [II], via the CoOP strategy.

Understanding and controlling ICD is an essential tool that plays a paramount role in advancing cancer immunotherapy and vaccine development. We demonstrated the controlled ICD mechanism of [II] in vitro with various cell lines and observed broadened IgG profiles: IgG1 and IgG2a in mice vaccinated with the [II] and influenza HA subunit. Peptide‐aggregate induced immune rupture (PAIIR) via [II] pairs (“pa[II]r”) has tremendous potential to advance healthcare and basic science applications based on immune system modulation.

## 4 Experimental Section

### Statistical Analysis

Pre‐processing of data did not include outlier exclusion. Data were analyzed by using GraphPad Prism Version 9.3.1. Figure preparation was done by using Adobe Illustrator 2021. Figures 3D, 3E, 4B, 4H and 6D were normalized to the mean of untreated control. Figure 5A and 6E were normalized to the mean of Triton‐X treated condition for LDH release (Maximum LDH release). At least three biological replicates were performed for in vitro studies, and *n* = 6 per group was performed for the vaccination study. All data are presented as mean ± standard deviation (SD). Statistical analysis was done by one‐way analysis of variance (ANOVA) with Tukey's multiple‐comparison test. **** p < 0.0001, *** p < 0.001, ** p < 0.01, * p < 0.1.

### Materials

9‐fluorenylmethoxycarbonyl (Fmoc) protected amino acids, [4‐[α‐(2',4'‐dimethoxyphenyl) Fmoc aminomethyl] phenoxy] acetamidonorleucyl‐MBHA resin (Rink amide MBHA resin), Oxyma, N,N'‐Diisopropylcarbodiimide (DIC), Trifluoroacetic acid (TFA), piperidine, dimethylformamide (DMF), dichloromethane (DCM) were purchased from Gyros Protein Technologies. Triisopropylsilane, Acetic anhydride, Congo Red dye and pyrene were purchased from Sigma‐Aldrich. Deionized water (resistance of 18.2 MΩ.cm) was used during the experiments.

### Cell Culture and Reagents

Epithelial ovarian cancer cell line OVCAR‐8 (NCI‐Vial Designation 0507715), B16F10, Panc02, 3T3 and THP‐1 cell lines were cultured in a humidified incubator at 37 °C supplied with 5% CO2. B16F10 and Panc02 cell lines were gifts from Dr. Wei R. Chen, University of Oklahoma. THP‐1 cell line was gift from Dr. Stefan Wilhelm, University of Oklahoma. OVCAR‐8, THP‐1, Panc02 cells a were cultured in Roswell Park Memorial Institute (RPMI) media (SIGMA R8758) and B16F10 and 3T3 cells were cultured in Dulbecco's Modified Eagle Medium (DMEM) (Sigma, D6429), supplemented with 10% FBS (Hyclone SH30910.03) and 1% antibiotics; penicillin (100 U mL^−1^), and streptomycin (100 µg mL^−1^) (Thermo Fisher 15240062) according to the manufacturer's instructions. T75 flasks (TPP 90076) were used to culture cells and cells were passaged upon 85% confluency by using trypsin (Sigma 59418C). The media was changed every 2 days.

### Synthesis and Characterization of FF Peptides

FF peptides (KFFAAK, EFFAAE, KFFWWK, EFFWWE, KFFIIK, and EFFIIE) were purchased from Biomatik (Biomatik Corporation, Canada) with higher than 95% purity. EFFVVE, EFFLLE, KFFVVK and KFFLLK peptides were synthesized via solid‐phase peptide synthesis with PreludeX automatic peptide synthesizer (Protein Technologies, Inc., Tucson, AZ). Peptides were prepared on a 0.2 scale by repeated amino acid couplings using Fmoc protected amino acid (3 eq.), DIC (7.5 eq.) and Oxyma (7.5 eq.). BHA Rink Amide resin was used as solid support to construct the peptides. Fmoc protected amino acids except the final residue were removed through treatment with 20% piperidine/DMF solution for 10 min (twice) at 50 °C. Cleavage of the peptides from resin and deprotection of acid labile protected amino acids were carried out with a mixture of TFA/TIS/water in a ratio of 95:2.5:2.5 for 2.5 h. Excess TFA and organic solvents were removed by solvent evaporator and the remaining peptide was precipitated using diethyl ether at 20 °C overnight. The centrifuged white peptide residue was dissolved in water and frozen at −80 °C overnight, and then lyophilized (Labconco Freezone, 12 L) for one or two days. Peptides were purified with preparative HPLC (Agilent 1260) and identified by Shimadzu LCMS‐2020. Agilent ZORBAX 300 SB‐C18 (9.4 x 250 mm) and Alltech Pro‐sphere HP C4 300A 5u (250 mm x 4.6 mm) with a mobile phase of water/acetonitrile mixture (0.1% am‐monium hydroxide) used for negatively charged peptides; water/acetonitrile mixture (0.1% formic acid) was used for positively charged peptides. All peptides were tested with a purity >95%. HPLC run started with 100% water for 3 min, followed by a gradient increase in acetonitrile from 0% to 60% over 30 min, followed by 100% acetonitrile for 3 min, and finally 100% water for 3 min. The flow rate is 1.5 mL min^−1^, and the injection volume is 10 µL.

### Peptide Aggregation Analysis

All peptide aggregation analyses were performed in PBS. DPH assay was performed to understand the CAC. Peptides (except [AA]) were prepared in PBS starting from a concentration of 500 µm to 3.9 µm with serial dilution. [AA] was prepared from 10 mm to 15.6 µm to check the aggregation behavior even in higher concentrations. First, negatively charged peptide (48 µL) was put to the black 96 well‐ plate, then 4 µL (from 4 µm in PBS) DPH was added, and finally, positively charged peptide (48 µL) was added to each well. The solutions were incubated at 37 °C for an hour. After the incubation, fluorescence intensity was collected immediately with Ex:360±40 nm, Em: 460±40 nm with BioTek Neo2SM microplate reader. We performed a Congo Red assay to visualize aggregations deposited on the well plate. Peptides having 0.5 mm concentration and equal volume (48 µL each) were prepared in PBS. Then first, negatively charged peptide was put to 96 well‐plate. Congo red having a final concentration of 20 µm (4 µL), was added, followed by the addition of a positively charged peptide (48 µL each). Brightfield images were taken immediately with Keyence bz‐x710 microscope at defined time points. Finally, ThT assay was performed for aggregation kinetics analysis. Peptides were prepared with the same method as the Congo Red assay, but the total peptide volume was adjusted to 196 µL. Final ThT concentration in peptide solution is 10 µm. Fluorescence measurements were taken immediately after putting positively charged peptide with Ex: 440±10 nm, Em: 480±10 nm.

### TEM Analysis

Peptide samples were prepared from 10 mm stock solutions (incubated for 24h) of co‐assembled samples to 0.02 mm with water. Then, 10 µL of diluted peptide samples were dropped onto the TEM grid (Ted Pella, Catalog number:01813‐F) and incubated for 10 min. Then, samples were taken with a pipette, negatively stained with 2% uranyl acetate and imaged using JEOL 2010F Field Emission Transmission Electron Microscope.

### Peptide Treatment and Viability Experiments

Positively and negatively charged self‐assembling peptides were mixed 30 min prior to experiment except where otherwise stated. The viability of the cell lines was measured by using CellTiter‐Glo 2.0 Reagent (Promega G9248). The luminescent signal was measured in accordance with the manufacturer's instructions. Measurements were carried out with BioTek Neo2SM microplate reader and relative viability was calculated. Live‐Dead assay was carried out using Viability/Cytotoxicity Assay kit (BIOTIUM 30002). After peptide treatment, cells were treated with calcein and ethidium homodimer in accordance with the manufacturer's instructions. Fluorescent images were taken by using Keyence bz‐x710 microscope.

### Induction of Different Cell Death Modalities

OVCAR‐8 cells were treated with 50 µm cisplatin for 24h to induce apoptosis. T75 flask containing OVCAR‐8 cells were incubated in a 50 °C water bath for 10 min followed by a 24 h recovery period for heat‐induced cytotoxicity. Three cycles of 3 min liquid nitrogen followed by 5 min of 37 °C incubation is used to induce F/T.

### Protein Extraction and Quantification–Lysate Proteins

RIPA lysis and extraction buffer (Thermo Fisher Scientific 89900) supplemented with halt protease & phosphatase inhibitor cocktail (Thermo Fisher Scientific 78440) is used to obtain lysate proteins. Supernatant proteins: Acetone precipitation is used for supernatant protein isolation. Briefly, ice‐cold acetone was added to supernatants (4:1, v:v), incubated at −20 °C for 60 min and centrifuged at 10 000 × g for 10 min. Acetone was removed and RIPA buffer was used to resuspend the proteins. Cell lysates were incubated for 15 min on ice and centrifuged at 14 000 × g for 15 min at 4 °C, and supernatants were collected according to the manufacturer's instructions. BCA protein assay kit (Thermo Fisher Scientific 23225) was used to quantify protein concentrations by measuring the absorbance at 562 nm in accordance with the manufacturer's instructions.

### Immunoblotting, Reagents

Acrylamide/bis‐acrylamide, 30% solution (Sigma A3699), 1.5 m Tris‐HCl, pH 8.8 (Teknova T1588), Tris HCl Buffer 0.5 m solution, sterile pH 6.8 (Bio Basic SD8122), Ammonium persulfate (Sigma A3678), UltraPure 10% SDS (Invitrogen 15553‐027), TEMED (Thermo Fisher Scientific 17919), Dithiothreitol (DTT) (BIO‐RAD 1610610) Tris Base (Fisher Bioreagents BP152), Glycine (Fisher Bioreagents BP381), 4x Laemmli sample buffer (BIO‐RAD 1610747), TWEEN 20 (Sigma P9416), Mini Trans‐Blot filter paper (BIO‐RAD 1703932), Nitrocellulose Membranes 0.45 µm (BIO‐RAD 1620115), Western Blotting Luminol Reagent (Santa Cruz sc‐2048). Antibodies; cleaved caspase‐3 (Cell Signaling, 9664S), caspase‐3 (Santa Cruz, sc‐7272). GSDME (Abcam, ab215191), β‐actin‐HRP (Santa Cruz, sc‐47778), HMGB‐1‐HRP (BioLegend, 651411), HSP90 (Santa Cruz, sc‐13119), Goat anti‐Rabbit IgG Secondary Antibody (Thermo Fisher Scientific, 31460), Goat anti‐Mouse IgG (Thermo Fisher Scientific, G‐21040)

### Procedure

Protein samples were diluted in Laemmli buffer and boiled for 5 min at 96 °C. Proteins were then separated by sodium dodecyl sulfate‐polyacrylamide gel electrophoresis (SDS‐PAGE) gels (8.5% and 15%) and transferred to nitrocellulose membranes. After transfer, the membranes were blocked in 5% milk in Tris Buffered Saline with 0.1% Tween 20 (TBST) or in 5% BSA for phosphorylated antibodies. Blots were then incubated with primary antibodies overnight. The next day blots were washed with TBST and incubated with HRP conjugated secondary antibodies. Lastly, western blotting luminol reagent solution was added on top of the membranes, and chemiluminescence signal was detected by Azure c600 Imaging Biosystems.

### LDH Assay

LDH release is measured by Cytoscan‐LDH Cytotoxicity Assay (Cat # 786‐210). Briefly, media from treated and untreated cells were collected at indicated time points, mixed with reaction mixture, and incubated at room temperature. The reaction was stopped using stop solution, and absorbance was measured at 490 nm, and 680 nm. Triton‐X treatment is used as a 100% LDH release positive control. Lastly, absorbance values at 680 nm (background signal) were subtracted from absorbance values at 490 nm and relative LDH release was calculated based on the positive control LDH release.

### Flow Cytometry

Cells were treated with either 0.25–0.5 or 1 mm [II] peptide for 6h and then stained with propidium iodide (Invitrogen, V13242) and analyzed by using BD Accuri C6 flow cytometry. Representative plots in the supporting information show PI‐positive populations. Percent (%) PI‐positive populations were plotted.

### Immunocytochemistry

Phalloidin staining OVCAR‐8 cells were seeded on glass coverslips in a 24 well plate. Cells were treated with [II] for 1‐3 and 6h. After each treatment period, cells were washed with 1X PBS and fixed with 4% PFA for 20 min, then, stained for 30 min with Phalloidin‐iFluor 555 (Abcam ab176756) in 1% BSA. After the staining coverslips were mounted in ProLong Glass Antifade Mountant with NucBlue Stain (Thermo Fisher P36981) and stored in the dark until imaging.

### Cytochrome c Staining

OVCAR‐8 cells were seeded on glass coverslips in a 24 well plate. Cells were treated with [II] for 1‐2‐3 and 4h. After each treatment period, cells were washed with 1X PBS and fixed with 4% PFA for 20 min. Then cell membranes were permeabilized by using Digitonin (0.002% in PBS) for 10 min on ice, blocked with 3% BSA in PBS, and stained with cytochrome c antibody in 1% overnight. The next day, samples were washed 3 times with PBS and incubated for 1 h at room temperature with Donkey Anti‐Mouse IgG NorthernLights NL493‐conjugated Antibody (NL009) in 1% BSA. Wells washed with 1% BSA and coverslips were mounted in ProLong Glass Antifade Mountant with NucBlue Stain (Thermo Fisher P36981) and stored in the dark until imaging.

### THP‐1 Differentiation

THP‐1 monocytes were differentiated in the presence of 200 ng mL^−1^ phorbol 12‐myristate‐13‐acetate (PMA) for 2 days. Then macrophages were incubated in serum‐free RPMI medium for 1 day prior to co‐culture studies. Prior to the co‐culture experiment, OVCAR‐8 cells were labeled with CellTracker Red CMTPX (Invitrogen, C34552) and THP‐1 differentiated macrophages were labeled with Wheat Germ Agglutinin (WGA‐488) (Biotium, 29022‐1). 0.5 mm [II] treated or untreated cells were transferred onto differentiated and 24h rested macrophages in a ratio of 1:1. Briefly 20.000 treated or untreated OVCAR‐8 cells were transferred to 20.000 THP‐1 differentiated macrophages and co‐cultured for 2h. Then, samples were fixed with 4% PFA, washed with PBS and mounted in ProLongTM Glass Antifade Mountant with NucBlueTM Stain (Thermo Fisher Scientific, P36981) and stored in the dark until imaging.

### Ethics

The study was carried out accordingly with the recommendations of Guide for the Care and Use of Laboratory Animals from the National Institute of Health. Animal procedures were approved by the OU Health Sciences Center (OUHSC) Institutional Animal Care and Use Committee (protocol number 20‐059‐CHI).

### Immunization and Sera Collection

Balb/c mice (*n*=6) were immunized subcutaneously with 5 µg of HA antigens, 0.5 mm [II] and their combination. Booster was done at D60, and sera were collected at D14, D28 and D75. 4 different influenza antigens; Recombinant HA protein from flu A/Brisbane/59/2007 (H1N1)(NR‐28607), HA from influenza A/New Caledonia/20/99 (H1N1) (NR‐48873), HA Protein from flu Virus A/St. Petersburg/100/2011 (H1N1) (NR‐34588) and Recombinant HA protein from flu A/California/04/2009 (H1N1) (NR‐15749) were obtained from BEI Resources. Peptides are mixed for 30 min as explained in the manuscript and mixed with the antigens at room temperature to formulate the vaccines before immunizations.

### ELISA

To measure antigen‐specific antibody responses, enzyme‐linked immunosorbent assay (ELISA) Nunc MaxiSorp flat‐bottom plates (Invitrogen 44‐2404‐21) were coated with 2.5 µg mL^−1^ with the mixture of four antigens in phosphate coating buffer (0.1 m Na2HPO4 in deionized water, pH 9.0) overnight at 4 °C. Next day, plates were blocked with 1% bovine serum albumin in phosphate buffered saline‐Tween (1X PBS, 0.05% Tween) for 2h at room temperature. Plates were then washed 4x with PBS‐T and incubated overnight at 4 °C with serially diluted sera collected from mice in PBS‐T. The next day, wells were washed 4x with PBS‐T and incubated for 1h at room temperature either with HRP‐IgG1 (SouthernBiotech 1070‐05) (1:4,000) or HRP‐IgG2a (SouthernBiotech 1080‐05) (1:4000). Wells were subsequently washed 4x with PBS‐T and developed with 2,2‐azinobis(3‐ethylbenzthiazolinesulfonic acid) (ABTS) (VWR 95059‐146) substrate for 5 min at room temperature. At the end of the incubation, the reaction was stopped with 10% SDS solution. Absorbance was measured at 405 nm to determine endpoint antibody titers.

## Conflict of Interest

A patent application for the technology described in this study has been submitted by the authors.

## Supporting information

Supporting Information

## Data Availability

The data that support the findings of this study are available on request from the corresponding author. The data are not publicly available due to privacy or ethical restrictions.
