# Supplementary material for: Peptide Aggregation Induced Immunogenic Rupture (PAIIR)
Source: Adv Sci (Weinh). 2022 May 22;9(21):2105868. doi: 10.1002/advs.202105868 (PMC9313945; doi:10.1002/advs.202105868)
Supplement: Supplementary file 1 — Supporting Information [file ADVS-9-2105868-s001.pdf]

Peptide Aggregation Induced Immunogenic Rupture (PAIIR)

Gokhan Gunay,[1] Seren Hamsici,[1] Gillian A. Lang,[3] Mark L. Lang,[3] Susan Kovats,[3,4] Handan Acar*[1,2]

1 Stephenson School of Biomedical Engineering, University of Oklahoma, Norman, OK, 73069, US
2 Stephenson Cancer Center, University of Oklahoma Health Sciences Center Oklahoma City, OK, 73104, US
3 Microbiology and Immunology, University of Oklahoma Health Sciences Center, Oklahoma City, OK, 73104 USA
4 Oklahoma Medical Research Foundation, Oklahoma City, OK, 73104 USA
Email address: hacar@ou.edu

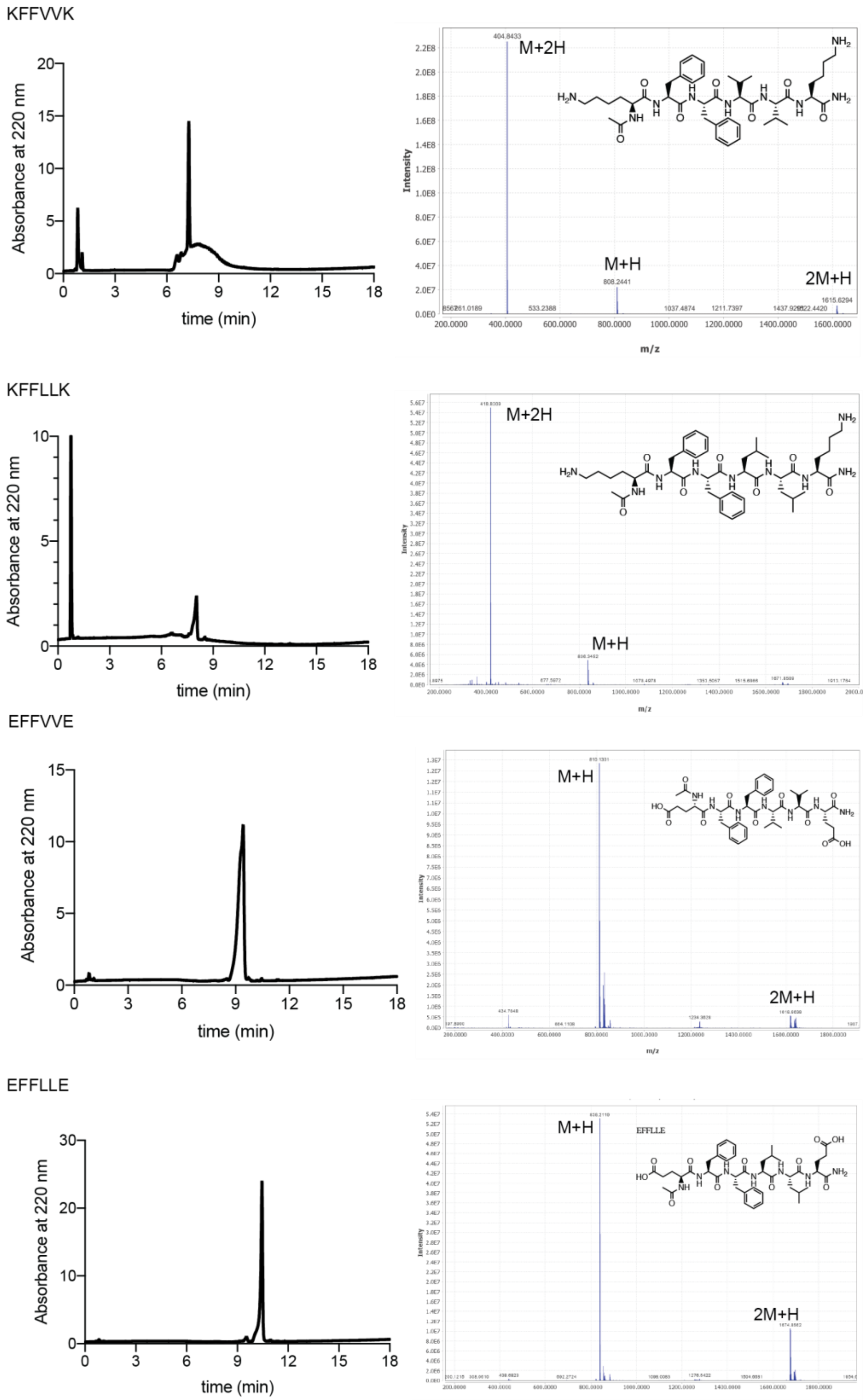

Fig. S1: **LC and MS analysis of LL and VV peptides**

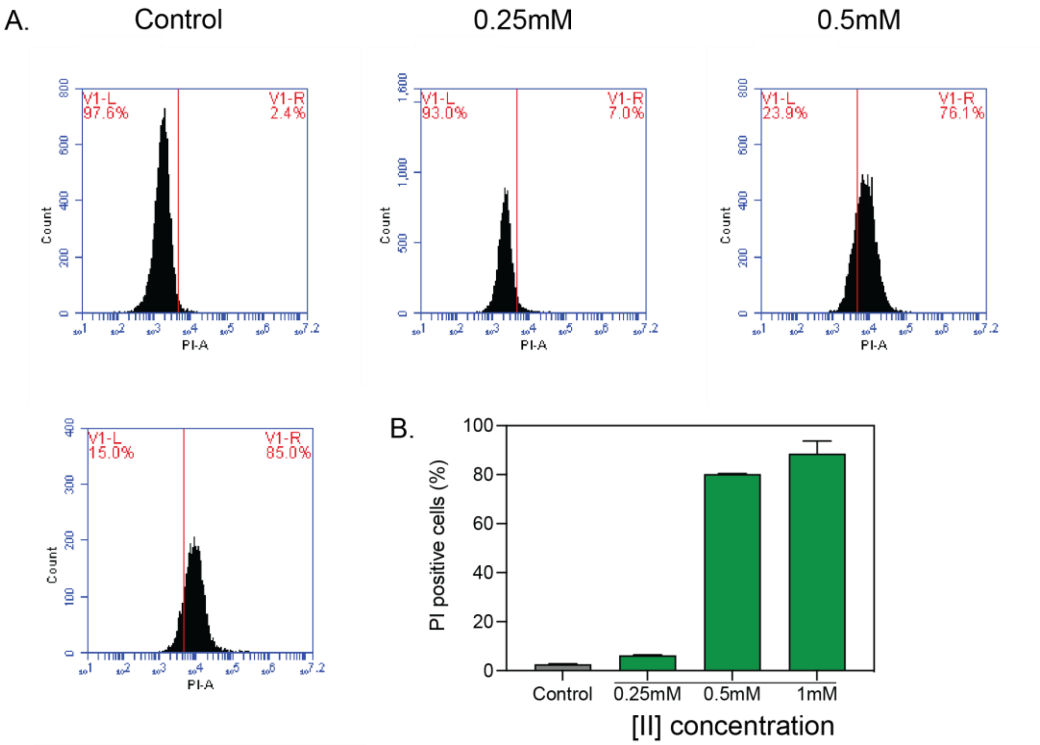

Figure S2: Propidium iodide (PI) uptake analysis by using flow cytometry. Representative histograms of untreated cells and cells treated with 0.25-0.5 and 1 mM [II] peptide for 6h (A). Percent (PI) positive cells at 6h.

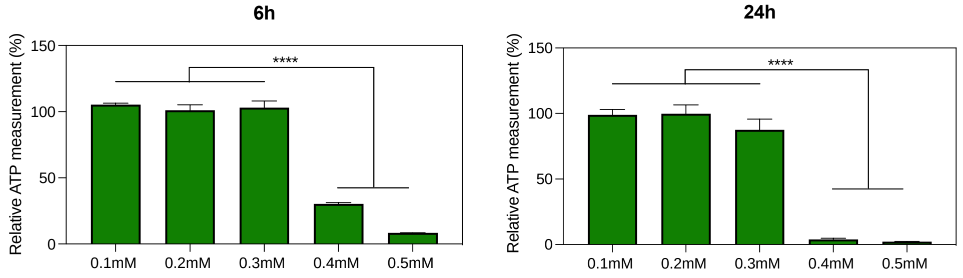

Figure S3: 6h and 24h cell viability measurements of OVCAR-8 cells treated with 0.1, 0.2, 0.3, 0.4 and 0.5 mM [II] peptide. Statistical analysis was done by one-way analysis of variance (ANOVA) with Tukey's multiple-comparison test. Data shown are mean ± SD, ****$p<0.0001$. n=3 replicates.

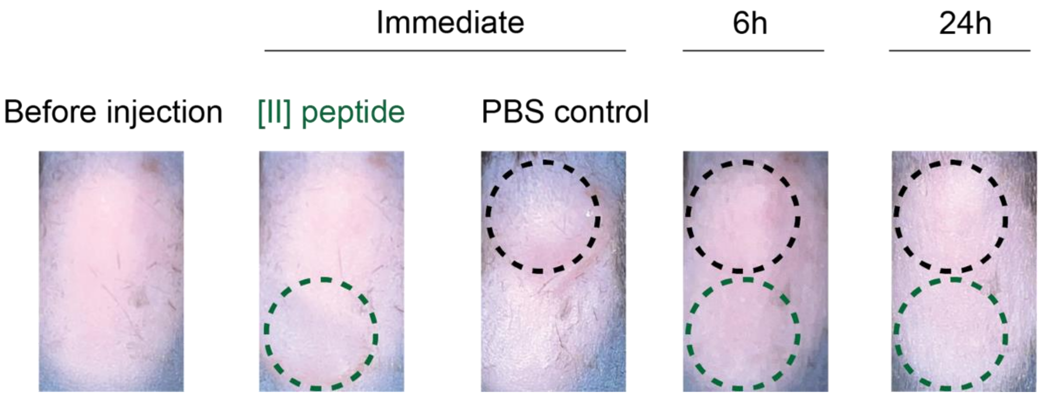

Figure S4: Effect of [II] peptide injection on skin. [II] peptide and PBS control were injected and skin was monitored immediately after injection followed by 6 and 24h.

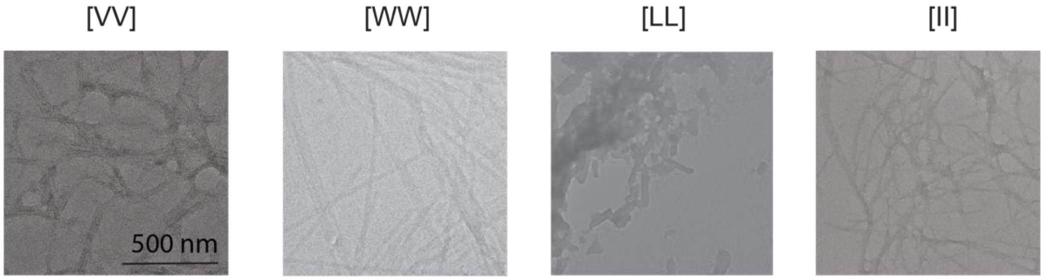

Figure S5: TEM images of [VV], [WW], [LL] and [II] peptides. Scale bar: 500 nm.
